# From Theory to Implementation: Adaptations to a Quality Improvement Initiative According to Implementation Context

**DOI:** 10.1177/10497323211058699

**Published:** 2021-11-12

**Authors:** Abimbola A. Olaniran, Modupe Oludipe, Zelee Hill, Adedoyin Ogunyemi, Nasir Umar, Kelechi Ohiri, Joanna Schellenberg, Tanya Marchant

**Affiliations:** 1 Department of Disease Control, 4919 London School of Hygiene and Tropical Medicine, London, UK; 2 Health Strategy and Delivery Foundation, Lagos, Nigeria; 3 Department of Community Health and Primary Care, College of Medicine, 98002 University of Lagos, Lagos, Nigeria

**Keywords:** quality improvement, maternal health, neonatal health, low- and middle-income countries

## Abstract

As countries continue to invest in quality improvement (QI) initiatives in health facilities, it is important to acknowledge the role of context in implementation. We conducted a qualitative study between February 2019 and January 2020 to explore how a QI initiative was adapted to enable implementation in three facility types: primary health centres, public hospitals and private facilities in Lagos State, Nigeria.

Despite a common theory of change, implementation of the initiative needed to be adapted to accommodate the local needs, priorities and organisational culture of each facility type. Across facility types, inadequate human and capital resources constrained implementation and necessitated an extension of the initiative’s duration. In public facilities, the local governance structure was adapted to facilitate coordination, but similar adaptations to governance were not possible for private facilities. Our findings highlight the importance of anticipating and planning for the local adaptation of QI initiatives according to implementation environment.

## Background

Despite reductions in maternal and neonatal mortality there continue to be a large number of avoidable deaths due to the poor quality of services for mothers and newborns in low- and middle-income countries (LMICs) (Kruk, Gage, Arsenault, et al., 2018). Improvement in access to care has not been accompanied by sufficient improvement in the quality of care provided, and poor quality of care accounts for a major proportion of maternal and neonatal deaths in LMICs (Kruk, Gage, Joseph, et al., 2018). In Nigeria, neither the maternal mortality ratio (MMR) nor the neonatal mortality rate (NMR) improved markedly during the era of the Millennium Development Goals: in 2008 and 2018, the MMR was estimated at 545 and 512 per 100 000 live births and the NMR at 40 and 39 per 1000 births, respectively (National Population Commission [NPC] & ICF, 2019; National Population Commission [NPC] & ICF Macro, 2009). Lagos State, the commercial capital of Nigeria where three quarters of women access health facilities for childbirth care, was estimated to have an MMR of 450 deaths per 100 000 live births in 2008 and NMR of 29 deaths per 1000 live births in 2016 (National Bureau of Statistics [NBS] & United Nations Children’s Fund [UNICEF], 2017; Oye-Adeniran et al., 2011), far higher than the Sustainable Development Goal country targets of MMR less than 140 deaths per 100 000 live births and NMR less than 12 births per 1000 live births by 2030 (World Health Organisation, 2014, 2020a).

Nigeria’s lack of progress may be partly explained by the lack of a clear strategy on healthcare quality, with no agency formally given the role of implementing and monitoring quality standards in healthcare delivery (HSDF, 2019). This lack of progress has prompted local and global stakeholders to broaden the focus from increasing health care access to also include quality improvement (QI) in service delivery (World Health Organisation, 2020b).

As interest in QI increases in LMICs, there are concerns that many examples of QI initiatives lack a detailed description of how changes were achieved and the role of context during implementation (Varley et al., 2020; Zamboni et al., 2020). Consequently, new implementers may not benefit from the knowledge of what worked, what was adapted, and why (Campbell et al., 2010; Siriwardena et al., 2014). A better understanding of the influence of context could improve the design, implementation and evaluation of QI initiatives, explain the mechanism of change and contribute to the programme theory for implementing QI initiatives in LMICs (Balbale et al., 2015; Coles et al., 2017; Sabot et al., 2018; Tancred et al., 2017; Zamboni et al., 2020).

Since 2015, the Lagos State Ministry of Health and the Primary Health Care Board have implemented the Nigeria Healthcare Quality Initiative (NHQI) to improve maternal and newborn health outcomes in Primary Health Centres (PHCs), public hospitals and private facilities (clinics and hospitals) in Lagos State. The NHQI was guided by the principles of QI described by the Institute for Healthcare Improvement (Institute for Healthcare Improvement [IHI], 2020) but contextualised to the Lagos health system and its facility types.

Seeking to contribute to the evidence base about how any why QI works, we investigated implementation of the NHQI intervention and how this was adapted in the Lagos health system.

## Methods

We aimed to explore the following two research questions: What adaptations were made to NHQI to enable implementation? How and why were these adaptations made?

### Study Design

A qualitative study was conducted using a multiple-case study design in which a case was defined as a facility type, that is, PHCs, public hospitals and private facilities. We combined an exploratory approach, which generates evidence using ‘what’ questions, and an explanatory approach that seeks to address ‘how’ and ‘why’ questions (Yin, 2009, 2014).

Through our study we wanted to understand the extent to which NHQI was adapted to fit the implementation context while also reflecting on whether and how contextual factors were adapted to enable NHQI. For this study, we defined context as a set of characteristics and circumstances that interacts, influences, modifies, facilitates or constrains an intervention and its implementation (May et al., 2016).

To guide our study, we hypothesised that important contextual factors would relate to the characteristics of the three different health facility types, represented by our three study cases, and features related to governance structures within the Lagos health system.

### Study Setting

In Lagos State, over 10,000 skilled health workers provide services to about 24 million people across a large number of facilities including three tertiary hospitals, 26 public secondary hospitals, 333 PHCs and 2886 private facilities (HEFAMAA, 2020). In Lagos, 27% of births take place in public facilities (tertiary hospitals, public secondary hospitals and PHCs), 48% in private facilities and 25% at home or other locations (National Population Commission [NPC] & ICF, 2019).

The Federal Ministry of Health governs two of the three tertiary hospitals, the third is governed by the state; the 26 public secondary hospitals are governed by the Health Service Commission of the Lagos State Ministry of Health; and the 333 PHCs are governed by the State Primary Health Care Board (Lagos State, 2006).

For this study, we focussed on the 50 facilities (six PHCs, 19 public hospitals and 25 private facilities) enrolled in November 2017 in the NHQI second phase which followed an initial phase that ran from April 2015 to September 2017. The NHQI leadership enrolled facilities based on (i) perceived will and commitment of leadership to engage in QI activities; (ii) high volume of maternal and neonatal cases; (iii) sufficient staff numbers to enable the formation of a QI team within the facility and (iv) availability of a data manager to organise and make facility health data accessible to the QI team.

### The Intervention and the theory of change

To understand intervention adaptations, it was first necessary to document planned processes. The NHQI aimed to reduce facility-based maternal and neonatal mortality and improve patient experience and satisfaction by addressing systemic issues that impede consistent delivery of quality health care. The NHQI theory of change illustrates the hypothesised pathway to change and defines the three QI activities that we focus on in this analysis (Supplementary file 1).

#### Establish collaboratives

The first activity was to establish functional and sustainable collaboratives for each facility type (three collaboratives in total) through political and financial commitment from key stakeholders. This entailed the formation of state and facility-level QI teams and the establishment of peer-to-peer learning platforms. The collaborative design drew on the Institute for Healthcare Improvement Breakthrough Series and the Model for Improvement framework which brings together QI team members from a large number of health facilities in ‘collaborative learning sessions’ to seek improvement in focused topic areas which are ultimately implemented in their respective facilities (Institute for Healthcare Improvement [IHI], 2020). It was projected that facilities would commit to working together for 18 months, alternating between ‘collaborative learning sessions’ and ‘action periods’. The ‘collaborative learning sessions’ were single-day biannual events that brought together all facility QI teams from the same facility type to engage in QI-focused peer-to-peer learning. The sessions were jointly facilitated by QI methodology experts from the state agencies and Health Strategy and Delivery Foundation (HSDF), a partner non-governmental organisation. Additionally, a mentorship programme was established to support QI activities and training in the longer term, enrolling enthusiastic facility QI team members for additional structured QI training. During the ‘action period’, facility QI teams were required to test change ideas that would help them achieve their improvement targets. The action period was supported by peer-to-peer learning through monthly cluster meetings (six PHCs forming one cluster and the 19 public hospitals forming four clusters) and WhatsApp groups. The private facilities had no mechanism for cluster meetings.

#### Build capacity

The second activity was to build the capacity of state-level stakeholders on governance and strengthen clinical and QI capacity of facility QI teams. Health Strategy and Delivery Foundation strengthened governance capacity by providing technical support to the state-level QI teams and private facility medical directors. The facility QI teams developed QI capacity during the biannual collaborative learning sessions, complemented with facility-based coaching and mentoring by HSDF and facility mentors.

#### Measurement and evaluation

The third activity was to strengthen measurement and evaluation structures by ensuring the availability of data, measurement tools and guidelines. A baseline assessment of state and facility readiness to implement QI was carried out and then each facility QI team was required to track performance on outcome and process indicators regularly. State-level stakeholders were expected to continuously review the progress of each collaborative based on aggregated facility data.

Overall, it was anticipated that these three activities would result in functional collaboratives and data management systems in addition to capacitated workers who could generate change ideas and provide quality services. In turn, these outputs were expected to result in competent care and systems, improved patient satisfaction and ultimately reduce facility-based maternal and neonatal mortality (Supplementary file 1).

### Data Collection

Data were collected for 12 months, between February 2019 and January 2020 and entailed a review of documents, key informant interviews with state and facility stakeholders and observation of collaborative learning sessions and cluster meetings. Except for one public hospital, the same facilities contributed data to the QI meeting reports and key informant interviews. Detail of data collection is described below and illustrated in Supplementary file 2.

1. Document review entailed an initial review of three NHQI documents to conceptualise the intervention’s design. Subsequently, meetings were held with implementers to seek clarification on the design. Additionally, 140 facility QI team reports were reviewed to gain an insight into the operationalisation of QI at the facility level.
2. Key informant interviews were conducted in English, with 45 participants purposively drawn from state and facility levels.

An initial list of government agencies and NGOs for interview was identified from early discussions with the NHQI primary implementer and the list grew to include other organisations based on discussions with other stakeholders and preliminary findings.

The staff of agencies or organisations were eligible if they played an active role in NHQI design or implementation or were involved in other projects with possible interactions with NHQI. Facility-level stakeholder selection was based on identifying more functional facility QI teams and team members, defined by evidence of regular facility QI team meeting attendance.

The study was introduced to potential participants through a single-page information sheet that included brief descriptions of the study and the email and phone number of the local research coordinators. The information sheet was shared via email and where possible hardcopies were delivered to potential participants. Interview dates and times were scheduled through follow-up emails or phone calls, based upon the availability of the potential participants. Three organisations invited to participate in the state-level key informant interviews declined participation.

Written informed consent was obtained from all participants. Using a topic guide, state-level participants were asked about the establishment of the collaboratives, capacity-building sessions, roles in measurement and evaluation, the support provided to NHQI facilities and how these influenced patient experience and outcomes. The topic guide for facility-level participants explored issues around the composition and operation of the facility QI teams and enablers and barriers of facility-level QI operation.

Data were collected until saturation was reached when additional data did not provide new information (Fusch & Ness, 2015). Saturation was established through an iterative process of preliminary data analysis during data collection in which it was noted that data from additional interviews continued to confirm emerging themes.

3. Seventeen observations were conducted at collaborative learning sessions and cluster meetings to gain an understanding of the variation in operation and priorities of collaborative sessions. Meetings for observation were identified through opportunistic sampling in which the researcher conducting the observations attended all the meetings he was aware of (Suri, 2011). For each meeting, the participants were notified that the session was being observed while the researcher made notes during the meeting. All observations were non-participatory such that the researcher did not play an active role in activities and discussions, thereby minimising the influence on the sessions and meetings.

### Data Analysis

Our thematic analysis (Boyatzis, 1998) entailed a combination of deductive and inductive approaches to data synthesis (Roberts et al., 2019; Ritchie et al., 2014). Using a deductive approach, a preliminary thematic map developed from the three key activities described in the theory of change guided familiarisation with the study data and entered into NVivo as the a priori themes. Subsequent analysis of transcripts was guided, but not confined, by the preliminary thematic map. Constructs within the data that explain implementation relating to the establishment of collaboratives, capacity building and measurement and evaluation were noted in inductive generation of the initial set of codes which were applied to the data on subsequent readings. The recurring patterns across congruent codes informed the subthemes (Ritchie et al., 2014). Identified subthemes were closely scrutinised for alignment with a priori themes and to ensure that they were representative of the codes and data.

To identify constructs relating to context, transcripts were reviewed against a priori themes on contextual influence identified from a limited literature review (Coles et al., 2020; Kaplan et al., 2012). The transcripts were further explored for the influence of these constructs in the implementation of the three key activities of NHQI. This informed an interpretive phase in which the roles of these constructs in implementation were developed into explanatory subthemes in the form of succinct phrases linking implementation with context.

Field notes from collaborative and cluster meetings were analysed using the same preliminary thematic map entered into NVivo for analysis of interview transcripts. Observation findings were used to (in) validate transcript findings and informed iterative revision of the topic guide to facilitate exploration of new emergent subthemes.

We triangulated across these multiple data sources to build trustworthiness: two researchers regularly reviewed and discussed the codes, analysis workshops were held with the larger team of researchers during and after the period of data collection and reflective notes were kept throughout data collection and analysis.

### Ethical Considerations

The study protocol was reviewed and approved by the Lagos State University Teaching Hospital Health Research Ethics committee (reference number-LREC/October 06, 1116) and the ethics committee of the London School of Hygiene & Tropical Medicine (ethics reference 16214). Before approaching interview participants, permission for data collection was obtained from the Lagos State Ministry of Health, Health Service Commission, Primary Healthcare Board and Medical Directors of the private facilities. Participation in the study was entirely voluntary, and participants could withdraw from the study at any time. Written informed consent was obtained from those who agreed to take part in the study.

## Results

This section reflects the adaptations made to each of the three activities defined by the NHQI theory of change (Supplementary file 1) and the critical role of governance, availability of resources and organisational culture in the adaptations.

### Establishment of Collaboratives

Establishment of three collaboratives entailed identifying governing agencies to oversee collaborative activities, selecting QI team members by participating facilities, and establishing physical platforms to aid learning and communication between state and facility level stakeholders. The extent of implementation of these activities varied by the characteristics of each facility type, the size of the collaborative and the adaptability of governance structures.

#### State-level governance structure was adapted and leveraged for coordination of QI activities but this structure was lacking in the private facility collaborative

An advantage of PHC and public hospital collaboratives was the existence of quality assurance teams at the State Primary Health Care Board and Health Service Commission of the Lagos State Ministry of Health which were adapted to govern the QI teams. Importantly, the presence of quality assurance teams in these agencies reflected political commitment to healthcare quality. A state-level participant explained how this was leveraged by ‘*forming QI teams [from existing quality assurance teams] within those agencies, and the QI teams would be able to oversee the quality work at the facility level’.* Lessons learnt from early stages of implementation revealed a hierarchical culture such that both governance and leadership were critical to establishing QI collaboratives. A state-level participant explained, ‘*…one of the key lessons was that the bottom-up did not really work… we had seen that we struggled with quite a number of facilities to implement quality improvement…We realised that if the management is the one driving QI, it is more likely to be sustained’.* Explaining the role of governing agencies in coordinating and supporting public facility QI meetings, a state-level participant said, ‘*So HSC [Health Service Commission: a governing agency], convenes cluster meetings…they sort out [fund] the logistics you know, transport logistics for people who are coming from the different hospitals [public hospitals], lunch…’*

In contrast, the professional body and accreditation unit considered for private facility collaborative governance did not have pre-existing teams with QI-related roles. A state-level participant explained, ‘*Initially for the private sector, we considered two bodies [a professional association of physicians and the accreditation unit of the state ministry of health] … but none of them had an existing structure for that [QI])’.* Particularly, the accreditation unit of the Lagos State Ministry of Health had limited human resources to form a QI team, and its primary focus was accreditation to inform facility license renewal based on quality assurance rather than QI. Furthermore, its retribution culture (placing sanctions on erring facilities) was considered inappropriate for the ‘no-blame’ culture of QI. Consequently, implementers needed to coordinate QI activities directly with the medical directors of all 25 private facilities and this negatively impacted cluster meetings and the mentorship programme. It was perceived that private facilities might be unwilling to co-fund meeting expenses or share information freely. The underlying competition between private providers and lack of leadership was seen to inhibit their collaboration. A state-level participant explained, ‘*For the private hospitals…we tested cluster [monthly cluster meetings] for like two or three months, and we knew they would not work because of the competition that exists in the private sector’*. This lack of collaboration between private facilities also negatively affected the QI mentorship programme and the potential for sustainability. Unlike PHC and public hospital mentors, the private facility mentors were only required to mentor their respective facilities as their medical directors were unlikely to permit support to other facilities. As explained by a state-level participant, ‘*… no MD [medical director] will let his staff still on his payroll take up how many hours to go for a meeting [mentors’ meeting] and then go to another hospital [mentor another private facility]’.* With no agency support, the mentorship programme in private facilities largely relied on retaining a QI mentor within facilities. As described by a state-level participant, ‘*our sustainability plan is our mentorship. It looks clearer in the public than in the private. Because in the public we have seen agencies to hinge the mentorship programme on, but in the private it’s hinged on individuals’.*

#### The size and membership of QI teams reflected varied facility size and hierarchical culture

A key adaptation to NHQI was QI team size and membership to accommodate different facility sizes while following a common team-selection guideline. A review of QI team meeting reports showed that team size ranged from as few as five members in a PHC to as many as 20 in a public hospital. Across facility types, the size and membership of QI teams reflected the number of departments and hierarchical culture in the health facilities. Accordingly, preference was given to heads of the department for departmental representation because of their decision-making power. A private facility QI team member stated, ‘*representatives from every department [departments in the health facility] are to bring the department to us, the problems in the department, the things that need to be improved and also take information back to them’.* A public hospital QI team member highlighting hierarchical culture explained that ‘*every head of department is part of the QI team so that the heads of the department can ensure that all the other staffs in their departments are following the guidelines’.*

#### Collaborative meetings were adapted to accommodate the relatively limited QI capacity of the PHC collaborative members

In contrast to the public hospital and private facility collaboratives, biannual collaborative learning sessions were not held for PHCs as it was observed that PHCs required more frequent contacts to address training needs, partly because of relatively high facility QI staff turnover rates. Alluding to the limited QI experience from high QI staff turnover rate, a state-level participant narrated, ‘*… we have health care workers [QI team members] from the PHCs … I don't think they are up to 12 months yet’.* Accordingly, it was considered that it would be feasible to bring six PHC QI teams together in monthly cluster meetings to build their capacity using the same collaborative learning session curriculum. A state-level participant explained, ‘*So, the PHCs …because they are only six facilities, so we can’t say we are having another learning session. So, since we are using the clustering method, we just stuck with it because they are only six facilities’.*

### Capacity Building

Different capacity building activities were designed for different levels: training for the state agency QI team and medical directors of private facilities was designed to focus on governance; while facility QI team training was designed to focus on QI activities, for example, identifying change ideas and using data to track progress. In reality, capacity building plans had to be adapted because of different facility priorities and also because of high staff turnover.

#### The content of facility QI team capacity building sessions was adapted to the remit of different facility types

There were notable differences in capacity building engagement according to the type of services provided by different facility levels. For example, eclampsia was noted as the leading cause of maternal deaths in the state in 2018 with most of the deaths occurring in public secondary facilities. A state-level participant explained that ‘*… So, for the public secondary, you will see that eclampsia was the leading cause of death. So, some private facilities did it, but we didn’t roll it out collaboratively because private facilities mothers were not dying… data didn’t show that it was a problem for them’.* The PHCs’ remit of basic and essential healthcare with the referral of emergencies to secondary level facilities also shaped the focus of the capacity building as explained by a state-level participant, ‘*the PHCs do not deal with secondary (complications/emergency) cases, so we just looked at strengthening things that have to do with primary health care’.*

#### The need for financial stability influenced the focus of private facility QI leadership training

Business and financial training content was built into the sessions of private facilities, reflecting the importance of securing adequate financial resources to fund the implementation of QI. A state-level participant narrated how some private facility medical directors were complaining that ‘*QI is taking away money from my hospital because you need to do some things right’.* During these leadership training sessions, the roles of medical directors in strengthening the business and financial management system of their respective facilities were emphasised. Subsequently, implementers worked with each facility to assess and address gaps in the facility’s financial management system. Justifying integration of financial management training into leadership training, a state-level participant explained, ‘*Because if you say QI takes away money from your hospital and then you are spending money on things that are not relevant. If we can plug those holes, perhaps you may have financial resources for QI’.*

#### Inadequate resources and high staff turnover across facility types necessitated longer engagement for capacity building

As NHQI progressed, it was observed that capacity building could not be completed within a fixed time frame but was a continuous process. High turnover of facility QI team members meant that additional capacity building sessions had to be programmed for new members. Furthermore, inadequate human resource capacity to implement QI activities, tools and commodities meant QI activities could not take place as planned, necessitating a longer period of engagement. Alluding to the resource gap after the first 18-month capacity-building period, a state-level participant stated ‘*… IHI breakthrough series is usually from 12 to 18 months.... I don’t feel that time frame is enough for Lagos… because if we are poor on input … it will take longer for you to achieve what you want to achieve, based on the resources at hand … the QI teams are changed. So, it wasn’t as if we were working with people that already understood, and we still had to do that training’.*

### Measurement and Evaluation

The measurement and evaluation activity for facilities entailed identification of indicators to track performance, tools to measure accountability and QI readiness, and routine analysis of data for decision-making. Public hospitals could respond easily to this activity but PHCs found it more difficult.

#### More measurement and evaluation tools were applied to the public hospital collaborative reflecting the governing agency’s political commitment to QI

Across all facility types, there were measures for assessing patient experience, tracking implementation of change ideas and viability of QI structure at the facility level. However, the public hospital collaborative had a more comprehensive set of tools, perhaps reflecting the political commitment of its governing agency to QI. Additionally, the governing agency of public hospitals was expected to conduct periodic analysis and presentation of data on facility performance to drive healthy competition between the hospitals. There were ongoing conversations on adapting measurement and evaluation tools to suit the PHC collaboratives but this was not achieved in the study period.

#### Lack of facility-based patient folders in the PHCs limited collaborative-level measurement and evaluation and decision-making

Across facility types it was observed that improvements in processes, for example, increased use of partographs, did not translate to improvements in maternal and neonatal mortality. In response, collaboratives decided to extract data on individual patient care from patient folders in an attempt to identify root causes of maternal and neonatal deaths and define collective QI responses. In the words of a state-level participant, ‘*So initially we were not prescriptive, we allowed facilities to come up with change idea by themselves, anything. But we found out that wasn’t moving the needle in terms of outcomes. … So, process indicators go up, and it becomes 80, 90% and you expect the outcome indicators to go down drastically as well…we focus on the outcome indicators, … using the data of the facility … brought all the HODs of O&G [Heads of department of Obstetrics and Gynaecology] together…we brainstorm together…they come up with what they think is the greatest problem, and they will come up with the change ideas…then we now roll it out across the collaborative’.* However, the PHC collaborative was not able to pursue in-depth root cause analysis because the patients went home with their case folders. A state-level participant explained, ‘*So, for the general hospitals and the private hospitals, they have facility records. So, it’s easy for us to audit their case note and get data. For the PHCs they didn’t have that, they didn’t have facility-based data’.*

## Discussion

All three core activities of the NHQI needed to be adapted to suit the implementation context in Lagos. Our findings underscore the importance of taking account of prevailing political commitment, the adaptability of available governance structures and the characteristics of facility types when planning QI implementation.

For example, the planned approach to strengthening the use of data for QI had to be adapted as implementers became more familiar with facility realities. PHCs had relatively little political capital, had limited availability of data, and struggled with measurement and evaluation activities. Private hospitals were prone to be guarded and may resist collaborative data sharing. Conversely, political interest in improvement in public hospitals resulted in close scrutiny of data there, transcending assessment of performance to also identifying and addressing facility-level human and capital resource gaps. Studies on QI across income settings underscore the relevance of measurement and evaluation systems in informed decision-making and highlight the benefits of generating data to enhance healthy competition through social pressure and reputational incentives (Adeniran et al., 2018; M. Dixon-Woods et al., 2011; Lighter, 2015; Russell et al., 2011). It has also been shown that data is key to understanding the complex interactions between an intervention and its implementation context, and how it translates to health outcomes (Ameh et al., 2017; Coyle & Battles, 1999; M. Dixon-Woods & Martin, 2016; Lavender et al., 2018; Mahdavi et al., 2018). Hence, funders seeking to invest in QI should assess and understand local stakeholders’ commitment to investing in and engaging with data. This assessment may guide adaptation of the intervention to include capacity-building of implementers on the use of data and/or advocacy to aid political commitment to data use that translates into an enabling context for QI implementation.

Similarly, we observed how NHQI was adapted to the varied collaborative size and facility resources. The relatively small size of the PHC collaborative, with just six facilities, was easier to manage, and enabled more frequent collaborative contacts, than the larger collaboratives. On the other hand, while the smaller PHC team permitted more focused engagement and coordination (Mao et al., 2016; Mueller, 2012; Shepperd, 1993), the larger hospital teams had a more diverse experience for addressing complex problems and for cross-facility, peer-to-peer learning (Goh et al., 2013; Mao et al., 2016; Weiss & Hoegl, 2016). A key barrier to implementation of QI activities in some facilities (irrespective of type) was the limited availability of resources, exacerbated by high staff turnover. Facilities with a high staff turnover needed more training sessions. Accordingly, inadequate structural resources directly limited the QI activities that teams could decide to engage in, obliging them to address these structural challenges first, before considering patient care. Overall, the combination of human resources for health and structural challenges meant the duration of NHQI had to be prolonged to accommodate these contextual challenges.

Our study also highlights components of the Lagos health system that could be adapted to accommodate NHQI implementation. In this study, existing governance structures within the state agencies were adapted and harnessed to enable a top-down and hierarchical state-level governance approach for PHC and public hospital collaboratives. Conversely, private facilities lacked a unifying governance structure thereby having relatively little support or coordination for collaborative functions, negatively impacting many of the QI activities. Nonetheless, there are inherent tensions between top-down and bottom-up approaches to governance with arguments for and against each. On the one hand, the top-down approach, that may be present in many organisational and societal cultures of LMICs, ensures staff compliance because of the fear of punishments from superiors. On the other hand, this approach does not encourage bottom-up ownership and problem-solving, potentially jeopardising sustainability (Coles et al., 2020; Zamboni et al., 2020). Where the precise parameters of governance structure are not amenable to change such as the top-down approach in LMICs, implementers may consider adaptation of the initiative’s strategies to the reality of the governance context.

This study has demonstrated the need to adapt the complex QI intervention to its implementation environment and it is important to acknowledge two consequences of this finding. First, that adapting content of an intervention to fit a context may compromise fidelity so that the intervention becomes difficult to package and replicate in other settings. Second, intervention adaptations that are needed because of shortfalls in the enabling environment, as may be true in a low-resource context, can negatively affect feasibility. The latter may explain why some interventions succeed in high-income but not low-income settings (May et al., 2016). Critiques of QI suggest that evidence of its effectiveness is mixed in part because of a tendency to replicate QI activities without considering local availability of supportive leadership, resources and culture, thereby ignoring the role of local context in influencing change (M. Dixon-Woods & Martin, 2016; Hulscher et al., 2013; Zamboni et al., 2020). Hence, there is a need for research to guide negotiation of an optimal balance between intervention fidelity to core concepts and adaptability to the environment of implementation, especially in LMIC settings.

### Study Limitations

This study makes key contributions to the existing body of knowledge on implementation of QI by examining adaptations made in different facility types of the same health setting. But there are study limitations. As in other qualitative studies, findings may not be generalisable beyond the study area, although many of the key findings appear to be consistent with literature from other settings. Non-functional QI teams were not included in key informant interviews, potentially missing insights to explain why some teams do not function. Furthermore, an impact evaluation was beyond the scope of this study thereby limiting the potential to attribute and consequently validate NHQI theory of change.

## Conclusion

Context plays a key role in the implementation of QI and the notion that successful initiatives implemented elsewhere will act as ‘plug-and-play’ solutions in a new setting is largely misguided. Over a period of five years in Lagos State, NHQI needed to be adapted to accommodate differences between the characteristics of PHC, public hospital and private facility types and their governance structures. To achieve the best possible gains from QI, our findings underscore the importance of being explicit about the likely influence of contextual factors on implementation and transparent about the need to commit time and resources to address them.

## Supplemental Material

sj-pdf-1-qhr-10.1177_10497323211058699 – Supplemental Material for From Theory to Implementation: Adaptations to a Quality Improvement Initiative According to Implementation Context Click here for additional data file. Supplemental Material, sj-pdf-1-qhr-10.1177_10497323211058699 for From Theory to Implementation: Adaptations to a Quality Improvement Initiative According to Implementation Context by Abimbola A. Olaniran, Modupe Oludipe, Zelee Hill, Adedoyin Ogunyemi, Nasir Umar, Kelechi Ohiri, Joanna Schellenberg and Tanya Marchant in Qualitative Health Research
