# Supplementary material for: From Theory to Implementation: Adaptations to a Quality Improvement Initiative According to Implementation Context
Source: Qual Health Res. 2021 Nov 12;32(4):646–55. doi: 10.1177/10497323211058699 (PMC8851672; doi:10.1177/10497323211058699)
Supplement: sj-pdf-1-qhr-10.1177_10497323211058699 – Supplemental Material for From Theory to Implementation: Adaptations to a Quality Improvement Initiative According to Implementation Context [file sj-pdf-1-qhr-10.1177_10497323211058699.pdf]

Supplementary file 1: NHQI theory of change

<!--Col Count:9-->Input		Activities		Output		Intermediate Outcome
**State leadership**<br><br>State QI champions.<br><br>**HSDF input**<br><br>6 Quality Improvement Officers.<br><br>6 Data analysts.<br><br>Prototype change packages.<br><br>Job aids.<br><br>Lean and six sigma training.<br><br>QI *how-to* tools.<br><br>**Health facilities**<br><br>Health care providers.<br><br>Equipment and supplies e.g. Bag and mask.<br><br>**Partner**s	Enablers: Effective partnership, adequate human and capital resources	**Establish Collaboratives**<br><br>Establish separate learning platforms for PHCs, public hospitals and private facilities.<br><br>Develop selection criteria for facility QI teams and allow the state to conduct selection.<br><br>Working with the state, establish QI teams in all health facilities.<br><br>Establish WhatsApp chat groups to facilitate continuous communication.<br><br>Test facility mentor-mentee approach.<br><br>**Capacity building**<br><br>Leadership and Facilitation training.<br><br>Training on data management for state and facility	Enablers: Motivated QI teams, quality training, dissemination of documents	**Collaboratives**<br><br>Clear roles of state-level QI teams.<br><br>Functional state and facility QI teams as evidenced by regular meetings.<br><br>Enhanced peer-peer learning platforms.<br><br>Protocol for facility mentor-mentee approach.<br><br>Facility advancement to graduation.<br><br>**Competent workforce**<br><br>State and facility-level QI teams with knowledge of QI methodology as evidenced by testing of local change ideas.<br><br>Clinically competent	Enablers: Effective collaboration between the QI team and other facility staff	**Strengthened anc responsive health system**<br><br>The institutionalisatio of QI in facilities a agencies.<br><br>Improved governance and accountability structures at the agencies.<br><br>Increased use of C knowledge and skills for decision making at the stat and facility levels.<br><br>**Improved quality care**<br><br>Improved matern and neonatal process indicators<br><br>Application of bes clinical practices.<br><br>Improved management of obstetric complications and neonatal ailments

<!--Col Count:9-->Input		Activities		Output		Intermediate Outcome
NURHI-PPFP<br><br>SMOH, HSC, HEFAMAA, PHCB QI teams.		staff.<br><br>Continuous QI capacity building for state and facility QI and data champions.<br><br>Strengthen the clinical capacity of healthcare providers.<br><br>Conduct monthly facility-based coaching and mentoring.<br><br>Train and support facility QI teams to identify and test change ideas.<br><br>**Measurement and evaluation**<br><br>Pre-baseline/baseline assessment.<br><br>Assessment of patient experience.<br><br>Analysis of monthly data.<br><br>Strengthening data management at the state level.<br><br>Conduct review		healthcare providers.<br><br>**Data and tools**<br><br>Baseline data, including patient experience.<br><br>Measurement SOP<br><br>Relevant job-aids and standardised materials for coaching and mentoring.<br><br>Dashboard and narratives.<br><br>Corrective action plans.		

<!--Col Count:9-->Input		Activities		Output		Intermediate Outcome
		meetings for performance assessment, accountability and dissemination.				

* Adapted with permission from HSDF. Nigeria Healthcare Quality Initiative (NHQI): Lagos Overview

Supplementary file 2: Overview of data collection*

<!--Col Count:6-->Data collection method	State (governmental and non-governmental)	PHC	Public hospital	Private facility	Total
**Document review**	3 documents on NHQI design and implementation	15 QI meeting reports (from 6 PHCs)	87 QI meeting reports (from 14 hospitals)	38 QI meeting reports (from 8 private facilities)	**143**
**Key informant interview**	12 (in 4 organisations)	9 (in 3 PHCs)	11 (in 4 hospitals)	13 (in 7 private facilities)	**45**
**Observation of meetings**		5 cluster meetings	-2 learning sessions<br><br>-6 cluster meetings	-2 learning sessions<br><br>-2 QI leadership training	**17**

*To protect anonymity a detailed breakdown of interviewees is not provided
